# Accurate Determination of Camera Quantum Efficiency from a Single Image

**DOI:** 10.3390/jimaging10070169

**Published:** 2024-07-16

**Authors:** Yuri Rzhanov

**Affiliations:** Center for Coastal and Ocean Mapping/Joint Hydrographic Center, University of New Hampshire, 24 Colovos Road, Durham, NH 03824, USA; yuri.rzhanov@unh.edu; Tel.: +1-603-7675572

**Keywords:** quantum efficiency, camera sensor characterization, color constancy

## Abstract

Knowledge of spectral sensitivity is important for high-precision comparison of images taken by different cameras and recognition of objects and interpretation of scenes for which color is an important cue. Direct estimation of quantum efficiency curves (QECs) is a complicated and tedious process requiring specialized equipment, and many camera manufacturers do not make spectral characteristics publicly available. This has led to the development of indirect techniques that are unreliable due to being highly sensitive to noise in the input data, and which often require the imposition of additional ad hoc conditions, some of which do not always hold. We demonstrate the reason for the lack of stability in the determination of QECs and propose an approach that guarantees the stability of QEC reconstruction, even in the presence of noise. A device for the realization of this approach is also proposed. The reported results were used as a basis for the granted US patent.

## 1. Introduction

The determination of camera spectral sensitivity (quantum efficiency (QE)) is important for many problems related to image acquisition. These problems include color correction for comparison of colors in images acquired by different cameras and under different illuminations, camera simulations [1], and sensor designs [2]. Another example problem is the reconstruction of the “true” color of an object imaged through an absorbing medium (for example, water), i.e., the reconstruction of the color that the object would have in the image if it were taken in air.

The “Gold Standard” colorimetric camera calibration procedure is described in [3,4,5]. This is a time-consuming procedure requiring expensive, specialized equipment and controlled conditions. However, even this procedure suffers from subjectivity. Acquired images lack spatial homogeneity; hence, the authors of Ref. [4] used averaging over a 21 × 21-pixel patch in the center of the image. Thus, although the QECs recovered by this technique are termed “ground truth”, the validity of this designation remains questionable.

Thus, it is not surprising that several approaches have been proposed to simplify the calibration procedure, such as utilizing an LED-based emissive chart [6], taking several images under arbitrary lighting conditions [7], or even taking just a single image of a multicolored target [8].

In these approaches, the image being processed is usually that of a standard reflective color target, such as the Gretag-Macbeth chart with known reflection spectra for each colored patch. Reconstruction of QECs is an ill-posed problem, as noted in [8,9], so the proposed techniques make use of additional constraints, such as the smoothness of the illuminant spectrum, fulfillment of Luther conditions, and non-negativity of QE functions. The ill-posedness of the problem is usually related in the literature to the limited dimensionality of the reflectance spectra [10,11,12].

For example, it was concluded that out of 1257 reflectance spectra from [13], only seven or eight are truly independent [10], and the rest can be constructed from the minimal set. From this conclusion, it follows that using only these seven or eight “almost” linearly independent spectra, QECs can be recovered at seven to eight wavelengths only, which is insufficient for practical purposes. This, in turn, leads to the conclusion that the Munsell chips and the Gretag-Macbeth chart are non-optimal choices for QEC recovery.

It was concluded that the optimal choice of 20 color samples gives almost as good a reconstruction of QECs as the use of all Munsell chips [12]. Those authors proposed to minimize the influence of noise by using the principal eigenvector (or rank-deficient pseudoinverse) solution. This paper also states that the sensor dynamic range plays an important role, and the increase of the range from the common 8 to 12 bits significantly improves the reconstruction. The simulation described by the authors has shown that the best root-mean-square error for spectral sensitivity estimation is 0.01796 (all 1269 reflectance spectra, 12-bit dynamic range).

The use of 16 glass dichroic transmission filters was proposed in [14]. The reconstruction of QECs required taking 16 images, cubic spline interpolation of the averaged measurements, and power correction. Note a strong overlap between filter transmission curves that led to distortion of the reconstructed curves. In this paper, it is shown that high overlap is the main cause of the reconstructed QECs’ distortion.

A spectrally tunable light source was employed for the same purpose in [15].

Recently, a comprehensive review of the spectral sensitivity estimation methods and a framework for QE estimation for consumer cameras was published [16]. However, the accuracy of the QEC recovery by the proposed approach remains questionable.

Currently, fast, reliable estimation of sensor QECs remains a problem for individual photographers and small companies lacking expensive equipment. It is worth noting that even cameras of the same make and model may have different QECs, as mentioned in [16]. The objective of this paper is to propose and describe a fast and accurate method for QEC determination.

## 2. Mathematical Formulation

To define notation for parameters, measured values, and spectral functions, the equations describing the color formation model for a trichromatic sensor and Lambertian shading conditions can be written as follows:

(1) $${\;v}_{f}=\int_{0}^{\infty }\omega I\left(\lambda \right)C\left(\lambda \right){\;s}_{f}\left(\lambda \right)d\lambda ,\;f∊\{r,g,b\},$$

where ${\;v}_{f}$ is a pixel value recorded by a color channel f, I(λ) is a light source depending on wavelength λ, ${\;s}_{f}\left(\lambda \right)$ is the sensor quantum efficiency, $C\left(\lambda \right)$ is a target reflectivity function (or spectral signature), and ω describes settable camera-related properties, such as gain, exposure time, etc. Effectively, integration is carried out over the visible range of the spectrum. By sampling spectral functions with the often-chosen ∆λ = 10 nm interval, the integral for a pixel i can be rewritten as a sum:

(2) $$v_{f,i}=\sum_{n=1}^{N}I\left(\lambda_{n}\right)C_{i}\left(\lambda_{n}\right){\;s}_{f}\left(\lambda_{n}\right)∆\lambda ,$$

where N is a number of samples with an interval ∆λ in the visible spectrum, and $C_{i}\left(\lambda_{n}\right)$ is the reflectance imaged at the pixel i. For M color patches, the known light source spectrum, and the patches’ reflectivity spectra, the above can be rewritten in matrix form, with ${\vec{F_{f}}}_{\;}={\left({\;F}_{f}\left(\lambda_{1}\right),{\;F}_{f}\left(\lambda_{2}\right),\ldots {\;F}_{f}\left(\lambda_{N}\right)\right)}^{T}$:

(3) $$\vec{V_{f}}=P{\vec{F_{f}}}_{\;},\;f∊\{r,g,b\}$$

The elements of the N×M matrix P consist of patches’ reflectivities for each dλ interval, and $\vec{F_{f}}$ is an element-wise product of I(λ) and $S_{f}$. P must be inverted (or pseudo-inverted, if M>N) to obtain three QECs. Due to this inversion being ill-posed, several techniques for obtaining sensible solutions have been proposed, such as Tikhonov regularization, Tikhonov derivatives-based regularization [17], linear models using some basis functions [9,12,18], and quadratic programming [19].

Note that M defines an upper bound for the number of samples N, and the greater the number of color patches used, the higher the spectral resolution of reconstructed QECs. Expecting a commonly accepted 10 nm resolution, no fewer than 31 different colors are needed for the 400–700 nm range and no fewer than 36 colors for the extended 380–730 nm range.

## 3. Previous Work and the Proposed Approach

The original Macbeth ColorChecker consists of 24 colored patches [20], which are chosen to represent “primary colors” and are “of general interest and utility for test purposes” [21]. The latest versions of charts manufactured by X-Rite have 140 or 240 patches. The reflectivity of these patches is known for the spectral range 380–730 nm with a 10 nm resolution. Increasing the number of different colors used in the QEC’s reconstruction process or choosing an “optimal” subset of colors does not improve the stability of the solution to Equation (3). The reason for this instability is the large condition number of the matrix P, as was already noted in [5]. Even minor perturbations of input data $\vec{V_{f}}$ lead to dramatic changes in recovered QECs. Whether all the Munsell colors are used or an optimally chosen subset of these colors, the condition number remains large, which guarantees instability in inversion.

To get a feel for the condition number value, 36 different random patches from the X-Rite ColorChecker were chosen. Repeating colors and glossy patches had previously been eliminated from consideration, leaving 189 different spectra. By increasing the number of random selections and keeping those with the smallest condition number, the latter saturates around the value of 31,000. According to [22], this means that matrix inversion leads to the loss of more than four digits of accuracy (in addition to the loss of precision due to the specifics of arithmetic methods and inaccuracy in input data measurements). In other words, the errors in the input data are multiplied by ~31,000, resulting in a significantly erroneous output. This leads to the conclusion that the reflectivity spectra of the X-Rite patches are not the ones that would allow for accurate QEC recovery.

**Figure 1:**
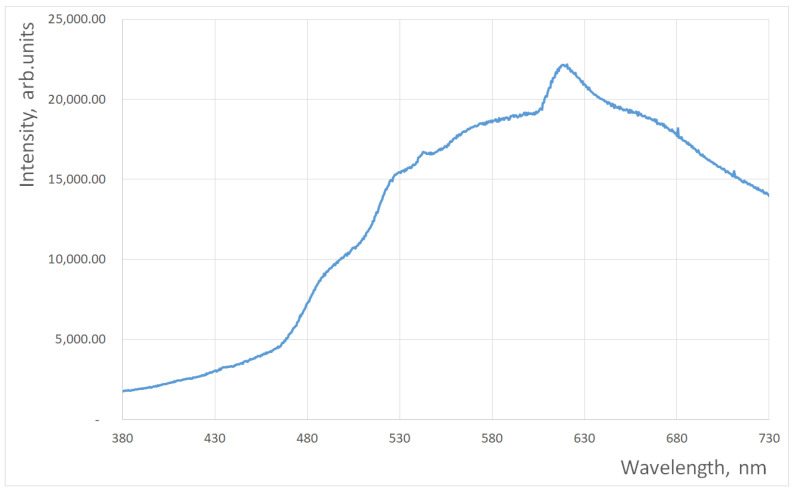
Spectrum of the illuminant used in simulations.

However, if the reflectivity spectra of patches comprise a disjoint set (only one reflectivity spectrum has a non-zero value at any wavelength), the condition number associated with the QECs’ recovery problem is exactly 1, and the precision of the solution is no worse than the input data (that is, measurements of the reflectivity spectra and RGB triplets). For the proof of concept, we conducted a numerical simulation based on Equation (3). To estimate the bandwidth of the spectra required for noise-tolerant QEC recovery, it is assumed that 36 reflective spectra have the Gaussian shape with the same standard deviation σ and maxima that are evenly distributed over the (extended) visible spectrum 380–730 nm, i.e., the number of Gaussians is L=36. Note that this number of spectra allows for the recovery of up to 36 points on each QE curve. For simulations, the standard spectrum of the incandescent lamp shown in Figure 1 was used, as were QECs found on the Internet for GoPro cameras (their exact shapes and the illumination spectra are irrelevant for the proof of concept). Colors recorded by a hypothetical camera were calculated using Equation (3) and scaled such that the maximum value for all colors and all color channels equals 255 (8 bits per pixel per channel).

Subsequently, the RGB triplets were corrupted by random noise, with the amplitude proportional to each value. Thus, K-percent noise changes the pixel value ρ to $\hat{\rho }=min(0,\max\left(255,\;\rho \times \left(1+R\frac{K}{100}\right)\right))$, where R is a random number in the [–1, 1] interval. All reported simulations used K=5 unless otherwise stated.

**Figure 2:**
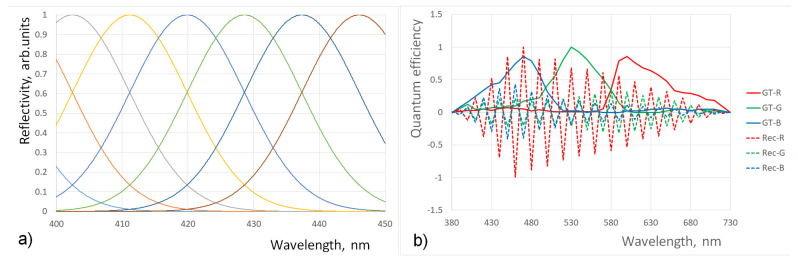
(**a**) Gaussian reflectance spectra in case of significant overlap; (**b**) comparison of ground truth (*GT*) and recovered (Rec) QECs for reflectance spectra shown in (**a**).

**Figure 3:**
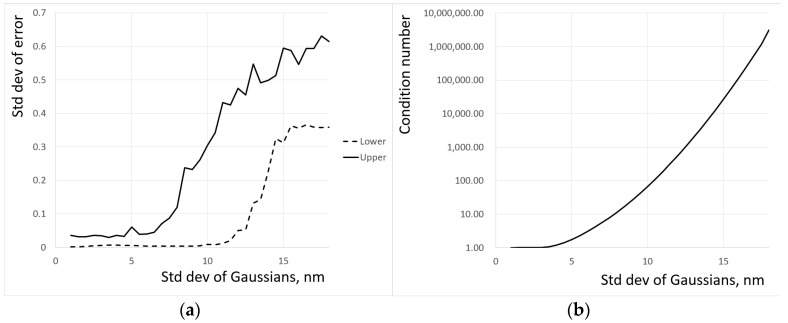
Reflectance spectra have a Gaussian shape. The errors in reconstructed QECs dramatically reduce when overlap between nearest Gaussians is approaching zero: (**a**) for the case of 36 measurements in the visible spectrum, the drop occurs between 10 and 7 nm; (**b**) the drop is directly related to the condition number of the matrix P. The condition number is approaching a value of 1.

Figure 2a shows the overlap of reflectivity spectra when Gaussians have a standard deviation of 15 nm. Figure 2b shows the deviation of the recovered QECs from the ground truth. The recovered QECs look much like those in [23]. The metric reflecting the quality of recovery is as follows:

(4) $$E\left(\sigma \right)=\sqrt{\sum_{k=1}^{L}{\left(Q_{k}^{GT}-Q_{k}^{R}(\sigma )\right)}^{2}}$$

where $Q_{k}^{GT}$ is the ground truth value of *QE* at the *k*-th wavelength and $Q_{k}^{R}$ is the corresponding recovered value. Reducing the standard deviation to 10 nm leads to an almost perfect recovery of the QECs. Figure 3a shows the dependence of $E\left(\sigma \right)$ on the standard deviation of the Gaussians, and Figure 3b shows the dependence of the condition number on *σ*. The non-monotonic behavior of the standard deviation of error is likely to be related to the randomness of the added noise. The main result of the simulation is that the reduction of the standard deviation of Gaussians representing the reflectivity spectra of color chips from 15 nm to 10 nm leads from nonsensical recovered QECs to almost perfect ones.

**Figure 4:**
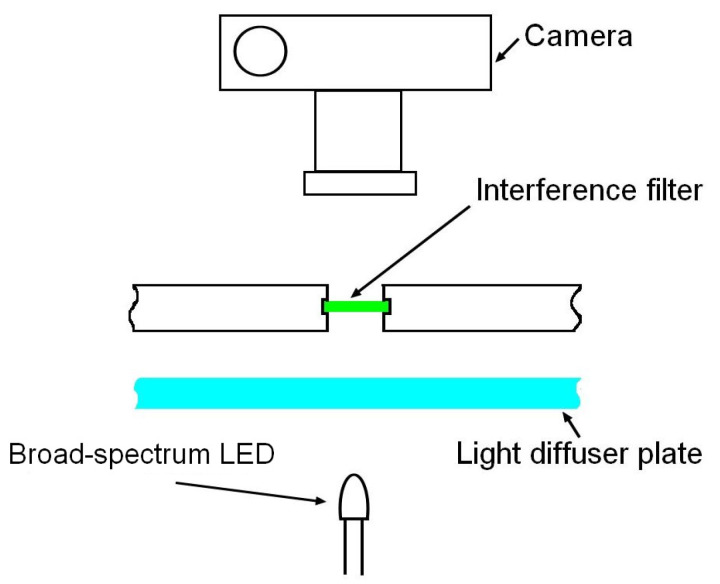
Proposed setup for a single cell with an interference filter. The complete device consists of 36 or 40 such cells. Detailed explanations are in the text.

However, the authors are not aware of paints or pigments with reflectivity spectra satisfying the criteria formulated above. In this paper, the use of transmitted light instead of reflected light is proposed. Interference filters with ultra-narrow transmission bands are available from many manufacturers. It should be noted that the use of interference filters for colorimetric calibration has been proposed in [11]. Those authors used a tungsten-halogen light to illuminate the standard ColorChecker through a set of broadband and narrowband interference filters. Photographs of the board and the spectral power distribution from each patch recorded by a spectrophotometer were then used to estimate the QECs of a camera. In this paper, the selection of a set of filters with non-overlapping bands and their illumination by a broadband light source through a diffusion plate for spatial homogenization are proposed. The transmitted light blob is then photographed. The typical sizes of filters are 1/2 inch and 1 inch. Forty filters assembled in an 8 by 5 array would have a size of approximately 16 by 10 cm. Thus, the use of a single light source is inconvenient due to the inhomogeneous illumination of different filters. The use of an array of identical LEDs, each back-lighting the corresponding interference filter (Figure 4), is proposed. Note that ambient light might substantially affect the accuracy of QEC recovery. Thus, the image must be taken in a dark room.

## 4. Algorithm for Estimating QECs

Summing up the proposed approach, one can outline the following algorithm:

1. Take a single image of an array of cells containing interference filters covering the entire visible spectrum (36 or 40 individual filters).
2. Calculate the average intensity for each cell around the brightest pixel. The choice of radius for averaging depends on the camera resolution but must be the same for all cells.
3. A vector of reflectivities (or transmissivities in this case) can be obtained from the known peak wavelength for each cell and its conversion to an RGB triplet (for example, as in [24]).
4. Matrix P. is (pseudo-)inverted. The inversion is stable as the matrix condition number is close to 1 (i.e., the matrix is almost diagonal).
5. The vector $\vec{F_{f}}$ is element-wise divided by known illumination intensity to obtain QECs for all three channels.

## 5. Proposed Implementation and Installation

**Figure 5:**
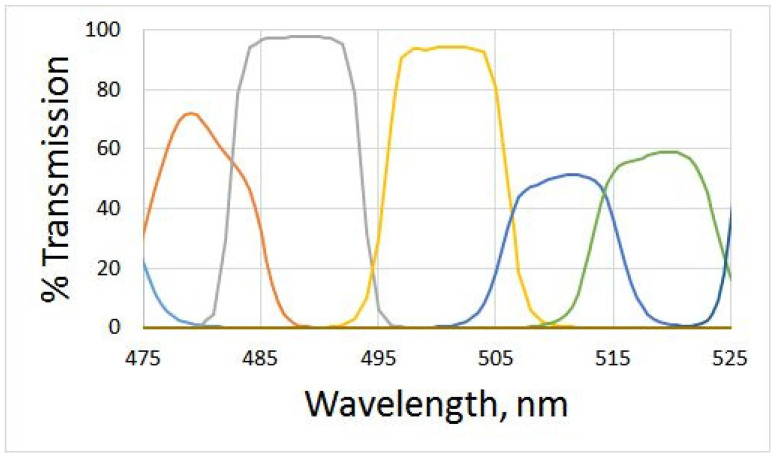
Transmission curves of some 10 nm filters manufactured by Omega Optical [26]. The nearest curves have some overlap.

**Figure 6:**
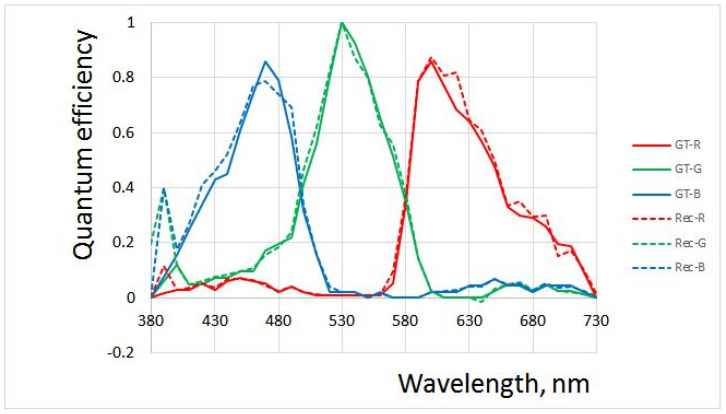
Difference between ground truth (*GT*) and recovered (Rec) quantum efficiency curves for 10 nm Omega Optical filters.

The most comprehensive sets of narrow band-pass interference filters are offered by Spectrogon [25], Omega Optical [26], Andover Corporation [27], and Thorlabs [28]. The transmission spectra of 10 nm FWHM filters manufactured by Omega Optical are published by the manufacturer, and some are shown in Figure 5. Simulations have demonstrated that recovered QECs have a sizable standard deviation of error E=0.051192 (i.e., around 5 percent) (Figure 6), which is consistent with the calculated value of the condition number 1237.71. The difference between the condition numbers calculated in the simulations described above and those of Omega Optical filters is due to the filters’ shape; the latter are far from having a Gaussian shape.

**Figure 7:**
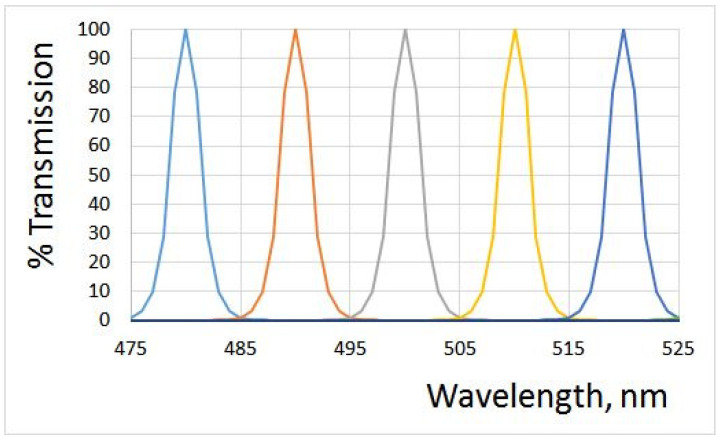
Transmission curves of some 3 nm filters manufactured by Andover Corporation [27]. The nearest curves have almost no overlap.

The ultra-narrow band-pass filters from Andover Corporation (Figure 7) (the website shows the parameters of the manufactured filters that were used in simulations) have FWHM 3 nm; their spectra have almost no overlap; the standard deviation of error E=0.00204, and the condition number is 1.003775. There is no visible difference between the ground truth QECs and the recovered ones.

**Figure 8:**
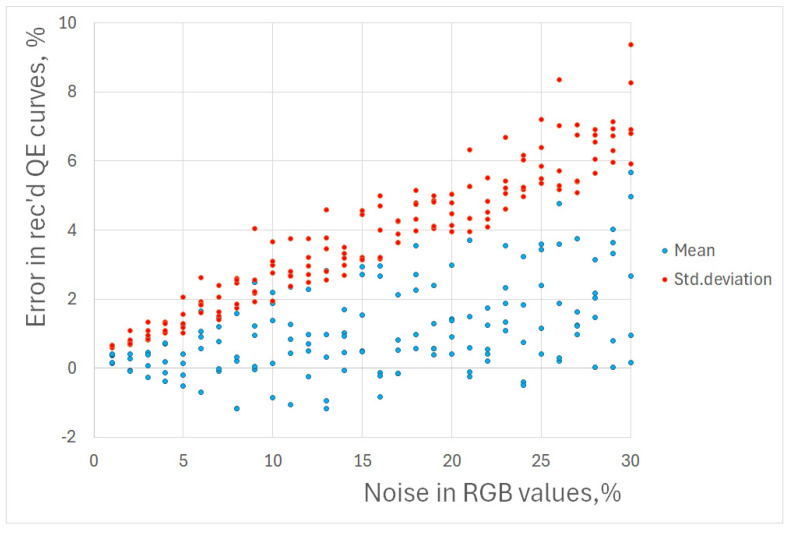
Dependence of mean and standard deviation of error in recovered QECs as a function of error in measured RGB triplets. Each simulation has been repeated five times.

Interestingly, the simulations indicate that even noisy measurements of RGB triplets lead to lower noise in recovered QECs, which is demonstrated in Figure 8. For an RGB triplet error of 15%, the standard deviation of error for QECs does not exceed 5%.

**Figure 9:**
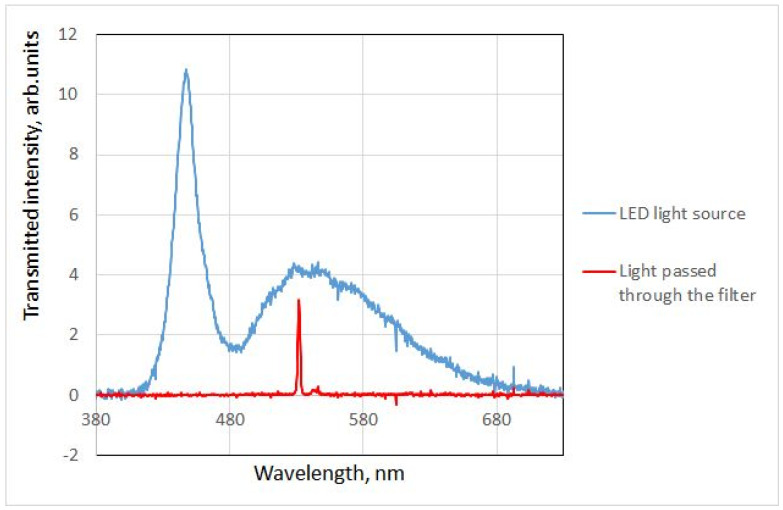
Spectra of light passed through the diffuser and through the diffuser and the filter.

Because a full set of filters (~36–40) is costly, it was decided to prove the concept with a single filter that was already acquired, specifically the interference filter with maximum transmittance at 532 nm and FWNM 3 nm manufactured by Thorlabs [28]. Using the setup shown in Figure 4, the spectra of light passing through the diffuser alone and through both the diffuser and the filter were recorded (Figure 9).

**Figure 10:**
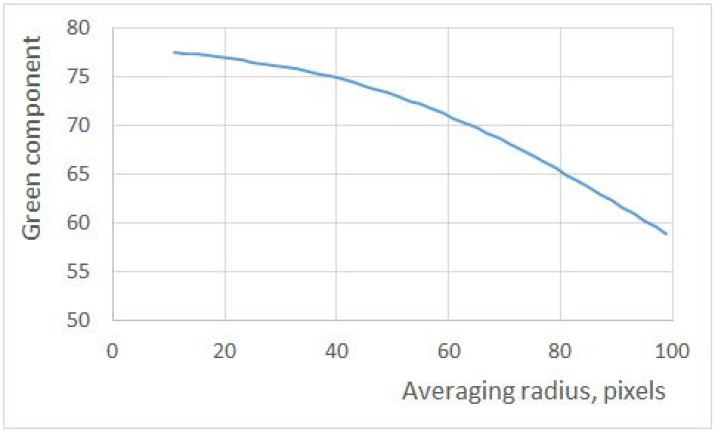
The dependence of the measured value in the green channel on the radius of averaging.

Due to the point light source, the illumination of both filters is spatially inhomogeneous, leaving uncertainty about how exactly the values of RGB triplets should be calculated. Figure 10 shows the dependence of the value of the green component (the two other components are nearly zero) on the radius of the averaged area. Note that this is essentially the same uncertainty that is present in [4]. As this bias is the same for all measurements and the recovered QECs are determined only up to a scale anyway, this uncertainty is not likely to affect the outcome. In our measurements, however, the illumination can be made spatially homogeneous with well-known techniques (for example, see [29]).

## 6. Discussion and Conclusions

We propose a technique and describe a device for determining the QECs for photo or video cameras using just a single picture. The main part of the device is a set of ultra-narrow band-pass interference filters. The spectra of these filters should overlap with each other as little as possible for reliable noise-tolerant recovery of QECs. The number of filters employed determines the number of wavelengths at which QECs are recovered. This suggests the use of filters with FWHM not exceeding 3 nm (and preferably with 1 nm FWHM) for maximally accurate recovery. The device can be used by manufacturers of imaging sensors and cameras and individual photographers for fast colorimetric calibration.

The numerical results given in the paper show that the main cause of inaccuracies in QECs’ reconstruction using images of colored chips is the input data noise amplification. Reduction of noise can be achieved by using a disjoint (non-overlapping) set of input data elements—in our case, signals from ultra-narrow band filters.

The proposed approach allows us to estimate QECs much more quickly than those approaches mentioned in introduction, as it only requires taking a single photograph. The “Gold Standard” technique produces the same results but requires at least 20–30 min to obtain 36 points on the QECs. To estimate QECs with the proposed technique, one needs to take a single image, which may take just a few seconds. The techniques that use images of colored chips are less accurate and often lead to the appearance of artifacts, as mentioned above.

The recent developments of pigments utilizing quantum dots [30] allow for the possibility of replacing interference filters with pigments with different properties. This direction of research has potential and deserves further investigation.

This work shows by means of numerical simulation that the use of ultra-narrow band interference filters allows for accurate reconstruction of camera QECs even in the presence of noise in the input data.

## 7. Patents

US Patent No. US11,202,062B2, titled “Methods and systems of determining quantum efficiency of a camera”, issued on 14 December 2021, claiming priority to the provisional application No. 62/589,104, filed on 21 November 2017.

## Data Availability

No new data were created or analyzed in this study. Data sharing is not applicable to this article.
